# ADP-ribose hydrolases: biological functions and potential therapeutic targets

**DOI:** 10.1017/erm.2024.17

**Published:** 2024-10-08

**Authors:** Jingpeng Wang, Zhao-Qi Wang, Wen Zong

**Affiliations:** 1 State Key Laboratory of Microbial Technology, Shandong University, Qingdao 266237, P. R. China; 2 Faculty of Biological Sciences, Friedrich-Schiller University of Jena, Jena 07743, Germany

**Keywords:** ADP-ribose hydrolases, ADP-ribosylation, PARPs

## Abstract

ADP-ribosylation (ADPRylation), which encompasses poly(ADP-ribosyl)ation and mono(ADP-ribosyl)ation, is an important post-translational modification catalysed by the poly(ADP-ribose) polymerase (PARP) enzyme superfamily. The process involves writers (PARPs) and erasers (ADP-ribose hydrolases), which work together to precisely regulate diverse cellular and molecular responses. Although the PARP-mediated synthesis of ADP-ribose (ADPr) has been well studied, ADPr degradation by degrading enzymes deserves further investigation. Nonetheless, recent studies have provided important new insights into the biology and functions of ADPr hydrolases. Notably, research has illuminated the significance of the poly(ADP-ribose) degradation pathway and its activation by the coordinated actions of poly(ADP-ribose) glycohydrolase and other ADPr hydrolases, which have been identified as key components of ADPRylation signalling networks. The degradation pathway has been proposed to play crucial roles in key cellular processes, such as DNA damage repair, chromatin dynamics, transcriptional regulation and cell death. A deep understanding of these ADPr erasing enzymes provides insights into the biological roles of ADPRylation in human health and disease aetiology and paves the road for the development of novel therapeutic strategies. This review article provides a summary of current knowledge about the biochemical and molecular functions of ADPr erasers and their physiological implications in human pathology.

## Introduction

ADP-ribosylation (hereinafter ADPRylation) is an evolutionarily conserved post-translational modification (PTM) process in which ADP-ribose (ADPr) is transferred from nicotinamide adenine dinucleotide (NAD^+^) onto specific amino acid residues (Asp, Glu, Arg, Ser, Lys, Cys) of target proteins primarily through the activity of members of the poly(ADP-ribose) polymerase (PARP) superfamily (Refs 1, 2). The mammalian PARP family consists of 17 members which catalyse either mono(ADP-ribosyl)ation (MARylation) or poly(ADP-ribosyl)ation (PARylation) (Ref. 3). MARylation, which is catalysed by the mono(ADP-ribosyl) transferases (e.g. PARP3, PARP4, PARP6–12 and PARP14–16), involves the covalent binding of a single ADPr molecule to the target protein. PARylation, which is catalysed by the poly(ADP-ribosyl) transferases (e.g. PARP1, PARP2, PARP5a and PARP5b), connects multiple ADPr molecules to form linear or branched poly-ADPr (PAR) chains (albeit PARP5a and PARP5b do not have branching activity) (Refs 3–5).

**Figure 1.**
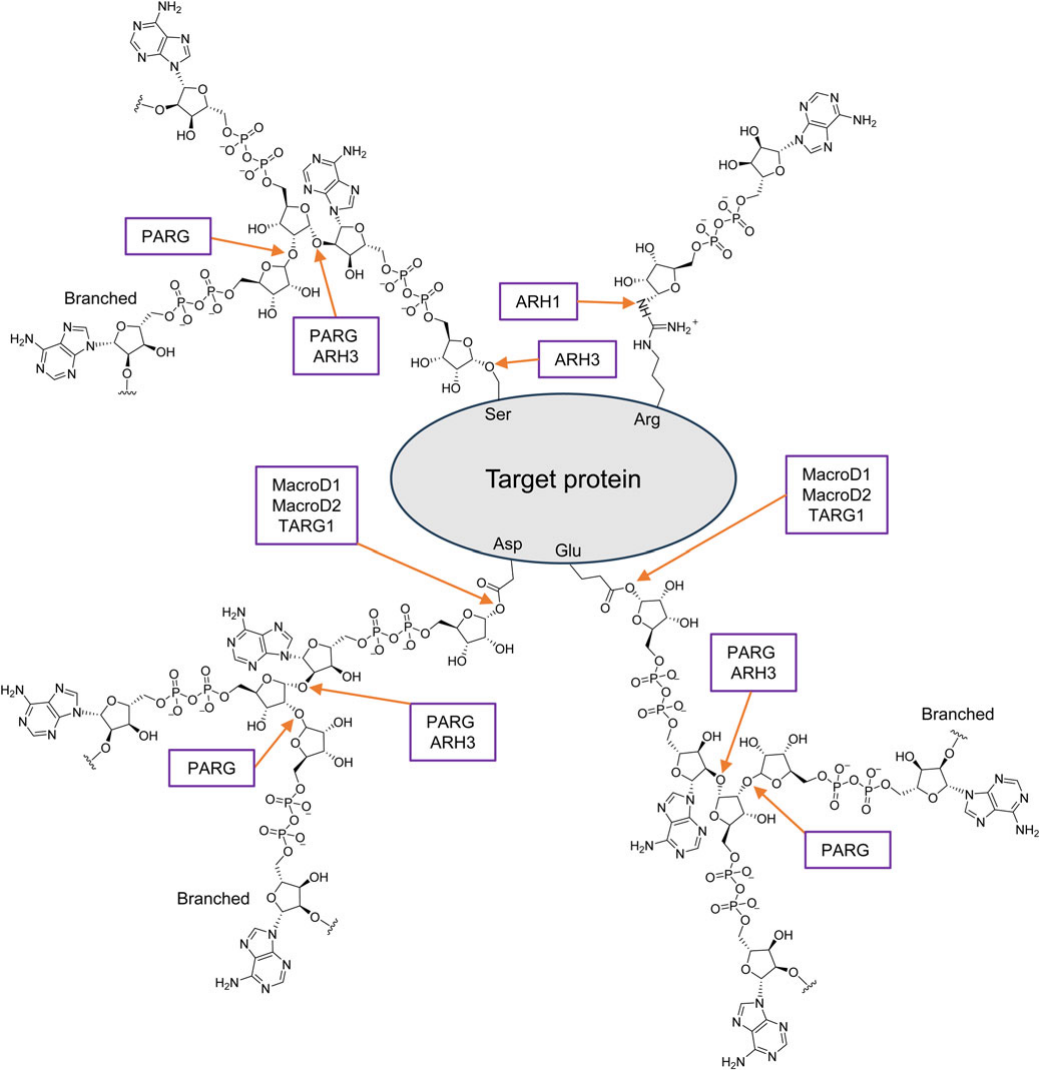
Catabolism of ADP-ribosylation. ADP-ribosylated proteins with bond-specific chemical cleavage sites for each ADP-ribose hydrolase. PARG is the primary poly(ADP-ribose) (PAR)-degrading enzyme, catalysing the glycosidic hydrolysis of the PAR chain. However, it is unable to cleave the last ADP-ribose moiety from mono(ADP-ribosyl)ated proteins. ARH3 catalyses the glycosidic hydrolysis of PAR chains, generating free ADP-ribose and short PAR chains. It also harbours hydrolysing mono(ADP-ribosyl)ation activity, specifically targeting *O*-linked ADP-ribosylation. ARH1 cleaves mono(ADP-ribosyl)ated substrates modified on arginine residues. MacroD1, MacroD2 and TARG1 hydrolyse mono(ADP-ribose) on the aspartate and glutamate residues of target proteins, and TARG1 can also cleave PAR chains.

Similar to other transient biological processes, the turnover of ADPRylation relies on both synthesis and degradation mechanisms. Both PAR and mono-ADPr (MAR) modifications on acceptor proteins in response to cellular and extracellular stimuli have been shown to be short-lived (Refs 6, 7). This rapid turnover underscores the importance of ADPr hydrolases in maintaining tight PAR homeostasis. In vertebrates, the hydrolysis of PAR or MAR is performed by members of two evolutionarily distinct families of ADPr hydrolases: the macrodomains and ADP-ribosyl-acceptor hydrolases (ARHs) (Ref. 8) (Table 1). A third family of microbial-derived ADPr hydrolases, known as the NADAR superfamily, has no known orthologues in vertebrates (Refs 9, 10). The macrodomain family members include macrodomain-containing proteins (MacroD1 and MacroD2), terminal ADP-ribose glycohydrolase (TARG1) and poly(ADP-ribose) glycohydrolase (PARG). The ARH family consists of three members (ARH1–ARH3), although ARH2 lacks apparent enzymatic activity (Ref. 11). The hydrolysis of PAR chains is performed mainly by PARG, whereas the much less-active ARH3 is speculated to serve as a PARG backup for this process (Refs 11–13). By contrast, ARH1 only hydrolyses MAR, mediating the release of ADPr from arginine residues of the target protein (Ref. 14). MacroD1, MacroD2 and TARG1 hydrolyse MAR at the glutamate and aspartate residues of the substrate, and TARG1 is also capable of cleaving PAR chains (Refs 15, 16) (Fig. 1).

**Table 1.** The family of human ADP-ribose hydrolases

ADP-ribose hydrolases	Alternative name	Mass	Class	Localisation	Substrate	Targeted residues
PARG	None	111/102/99/60/55 kDa	Macro	Nucleus, cytoplasm, mitochondria	PAR	None
TARG1	OARD1	17 kDa	Macro	Nucleus, cytoplasm	MAR/PAR	Asp/Glu
MacroD1	LRP16	35 kDa	Macro	Mitochondria	MAR	Asp/Glu
MacroD2	None	47 kDa	Macro	Nucleus, cytoplasm	MAR	Asp/Glu
ARH1	None	39 kDa	Ribosyl_crysJ1	Cytoplasm	MAR	Arg
ARH2	None	40 kDa	Ribosyl_crysJ1	Cytoplasm	Unknown	Unknown
ARH3	None	39 kDa	Ribosyl_crysJ1	Nucleus, cytoplasm, mitochondria	MAR/PAR	Ser

*Note*: Five PARG isoforms have been reported in human and have distinct subcellular localisation. PARG_111_ is localised in the nucleus, PARG_102_ and PARG_99_ are localised in cytoplasm, PARG_60_ is localised in cytoplasm and mitochondria, PARG_55_ is localised in mitochondria. The hydrolysis of MAR or PAR by ARH2 has not been demonstrated owing to the absence of critical amino acid residues necessary for enzymatic activity. PAR, poly(ADP-ribose); MAR, mono(ADP-ribose); Macro, microdomain; Ribosyl_crysJ1, ADP-ribosylation/Crystallin J1 fold.

ADPRylation homeostasis is vital for ensuring normal cellular activities. PAR metabolism has been well studied, mainly through research on the biological functions of PARPs and their inhibitors (PARPi), with great progress made in clinical applications of the latter (Ref. 17). Nevertheless, mounting evidence suggests that regulated MARylation also contributes to a wide range of cellular events, including endoplasmic reticulum and genotoxic stress, cellular metabolism and infection (Ref. 18). Because many informative excellent review articles have already focused on the functions of ADPr-synthesising protein families (Refs 19–24), we instead summarise herein recent findings on the roles of several ADPr hydrolases (PARG, ARH1, ARH3, MacroD1, MacroD2 and TARG1) in biochemical and physiological processes as well as the progress made in developing their corresponding inhibitors as potential pharmaceutical interventions.

## Poly(ADP-ribose) glycohydrolase

Poly(ADP-ribose) glycohydrolase (PARG), originally identified in a calf thymus nuclear preparation (Ref. 25), is the primary hydrolase involved in PAR chain degradation, being 1–2 orders of magnitude more active than ARH3 in this regard (Ref. 26). In mice, a single gene encodes two main PARG isoforms: 60 kDa (localised in the cytoplasm and mitochondria) and 110 kDa (in the nucleus) (Refs 27–29). By contrast, humans have five PARG isoforms: 55 kDa (in the mitochondria), 60 kDa (in the cytoplasm and mitochondria), 99 kDa (in the cytoplasm), 102 kDa (in the cytoplasm) and 111 kDa (in the nucleus) (Refs 29–32) (Table 1). The large human PARG isoforms (99, 102 and 111 kDa) are known to be yielded through alternative splicing (Ref. 30), whereas it remains unclear whether the short PARGs (55 and 60 kDa) result from degradation of the 111 kDa protein or alternative splicing.

**Figure 2.**
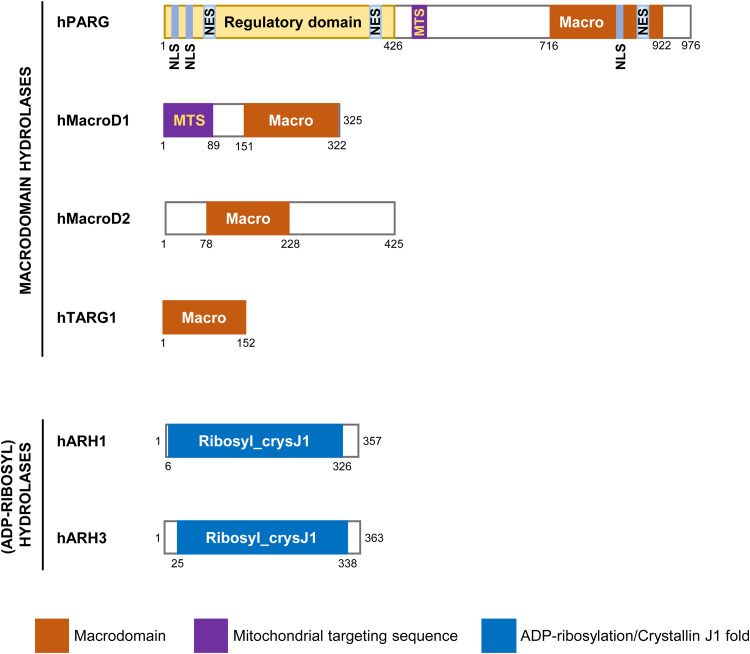
Schematic diagrams of human ADP-ribose hydrolases. The hydrolytic domains are Macro (macrodomain) and Ribosyl_crysJ1 (ADP-ribosylation/Crystallin J1 fold). No reports related ARH2 structure has been published. The N-terminal putative regulatory domain of PARG consists of two nuclear localisation signals (NLSs) and two nuclear export signals (NESs). The catalytic C-terminal domain contains one mitochondrial targeting sequence (MTS), one NES and one NLS. The N-terminal region of MacroD1 also contains one MTS.

In both humans and mice, the *PARG* gene consists of 18 exons. The full-length PARG protein comprises a putative regulatory domain (1–426 amino acids, encoded by exons 1–3) at the N-terminus and a conservative catalytic domain (486–838 amino acids, encoded by exons 9–14) at the C-terminus (Refs 29, 31) (Fig. 2). One study found that the existence of a mitochondrial targeting sequence (MTS) is the basis to ensure the catalytic activity of PARG, since the mutation of hydrophobic leucine residues in this element led to inactivation of the 59 and 111 kDa isoforms (albeit the specific mechanism behind this is unknown) (Ref. 33). In human PARG, two primary *α*-helical sub-domains flanking a twisted, mixed, 10-stranded *β*-sheet core are arranged to form a central cleft above the *β*-sheet in the catalytic domain, whereas in the mouse, the enzyme has one mixed nine-stranded *β*-sheet (Refs 34, 35). The deep cleft is the main ADPr-binding site and catalytic centre. After PARG binds to ADPr, the adjacent *β*_12_–*α*_10_ loop moves in a concerted manner near the binding site, and the Phe-902 side chain rotates to stack against the adenine moiety, which is secured by a network of direct and water molecule-mediated hydrogen bonds (Ref. 34). Glu-755 and Glu-756 (Glu-748 and Glu-749 in mice; Glu-114 and Glu-115 in bacteria) are the key catalytic residues (Refs 34–37). The critical ribose–ribose *O*-glycosidic linkage at the PAR terminal position is in direct hydrogen-bond contact with Glu-756, which then protonates the ribose' 2′-OH leaving group (Refs 34, 37). A tightly bound water molecule, positioned by interactions with Glu-755 and Asp-737, is activated through the concomitant protonation by Glu-756 and attacks the oxocarbenium intermediate, resulting in the release of ADPr and short unbranched PAR chains (Refs 34, 37).

### Biological function of PARG

#### Importance for animal development

PARG is the most well studied of the ADPr hydrolases. Its complete absence resulted in the death of *Drosophila melanogaster* larvae at the normal incubation temperature of 25 °C (Ref. 38). When the developmental temperature was increased to 29 °C, 25% of the mutants were able to develop into adulthood, albeit they only survived for approximately two weeks compared with the more than 1 month survival time of wild-type flies (Ref. 38). These surviving flies showed excessive PAR accumulation in the central nervous system as well as progressive neurodegeneration with reduced locomotor activity (Ref. 38), indicating the importance of PARG-mediated PAR degradation in the nervous system. Additionally, the phosphorylation of PARG by casein kinase 2 affected *Drosophila* larval development, with the loss of PARG phosphorylation reducing insect survival from the egg to adult stages (Ref. 39). In mice, the deletion of all PARG isoforms resulted in early embryonic lethality, with blastocytes accumulating PAR and subsequently dying (Refs 40, 41). Although the deletion of PARP1 partially rescued the PARG-deficient embryos, *Parg*/*Parp1* double-knockout mice which survived postnatally exhibited growth retardation and severe kidney failure and died within 3 months of birth (Ref. 40), indicating an essential role of ADPRylation homeostasis under the physiological status. However, the promoter sequence of the translocase of inner mitochondrial membrane 23 (*Timm23*) gene was additionally deleted in this *Parg*-knockout mice, thus reducing *Timm23* expression (Ref. 40). Therefore, whether the kidney failure was caused by PARylation imbalance or Timm23 disruption remains unclear. Interestingly, hypomorphic mutant mice lacking the 110 kDa PARG isoform are viable and fertile, suggesting that the 60 kDa isoform may sufficiently compensate for PARG activity in essential processes (Ref. 28). These studies indicate that PARG activity is essential for the development of organisms.

#### Gametogenesis

Two isoforms of PARG (60 and 110 kDa) are present in rat germinal cells, with the 110 kDa protein being predominantly present and active in the nuclear fraction of primary spermatocytes and the 60 kDa isoform abundant in the cytoplasmic fraction of round sperm cells (Ref. 42). The different intracellular distributions of the PARG isoforms indicate their possible roles in meiosis and post-meiosis. Loss of 110 kDa PARG resulted in decreased fertility in mice and reduced chromatin integrity in their sperm cells, although it did not affect sperm motility (Ref. 43). In germ cells of *Caenorhabditis elegans*, depletion of PARG2 (the orthologue of mammalian PARG) rendered the cells sensitive to ionising radiation and induced the over-expression of exonuclease 1 (EXO1) after DNA double-strand break (DSB) formation, leading to excessive end resection at the DSBs (Ref. 44). The resultant DNA intermediates with a long stretch of single-stranded DNA could not be processed by homologous recombination (HR), the main DSB repair pathway. The repair of DNA intermediates through the highly error-prone alternative end-joining pathway likely caused the high embryonic death rate after ionising radiation treatment (Ref. 44). PARG1, another *C. elegans* orthologue of mammalian PARG, promotes meiotic DSB formation and repair in a manner independent of its catalytic activity, ensuring the correct progression of germ cells (Ref. 45). The simultaneous depletion of PARG1 and the E3 ubiquitin ligase BRC1 resulted in decreased crossover formation and impaired DNA repair, leading to gamete death (Ref. 46). These findings indicate the role of PARG in the repair of DSBs, which are essential for spermatogenesis or general gametogenesis. Moreover, in *Drosophila*, the phosphorylation of PARG regulates the differentiation of germline stem cells into cystoblasts (Ref. 39). Taken together, these results identify the critical role of PARG in spermatogenesis or germ cell development as well as the reproductive process.

#### Stress response and cell death

Because excessive PAR accumulation can cause cell death, PARP1/2-mediated PARylation is a transient process, being eliminated by PARG in a short period (Ref. 7). The loss of PARG was shown to cause defects in the repair of single- and double-strand breaks and increase the radiosensitivity of the cells (Ref. 47). At the same time, the irradiated PARG-deficient cells exhibited centrosome expansion and mitotic defects, which induced polyploidy or cell death (Ref. 47). In embryonic trophoblasts, the lack of PARG caused compromised cell proliferation, PAR accumulation of histones, sensitivity to DNA damage agents and increased cell death (Refs 41, 48). Low PARG activity in neuronal cells also increased their death and sensitivity to *N*-methyl-D-aspartate, an excitotoxic inducer for neurons (Refs 49, 50). Hypomorphic mutation of PARG (i.e. lacking the 110 kDa isoform) rendered mouse embryonic fibroblasts (MEFs) hypersensitive to the DNA damage agents *N*-methyl-*N*-nitro-*N*-nitrosoguanidine and Adriamycin and increased their susceptibility to death (Refs 51, 52). The absence of PARG results in the excessive accumulation of PAR, which can lead to parthanatos, a cell death process that occurs when PAR binds to apoptosis-inducing factor (AIF) on the cytosolic side of the mitochondrial outer membrane, thereby interrupting binding of the factor to the mitochondria, which is lethal (Refs 53–56). Unlike traditional apoptosis, parthanatos does not involve caspase activation or the formation of typical apoptotic bodies (Ref. 56). Instead, the process relies on PAR and is hyper-stimulated by the release of AIF from mitochondria to elicit nuclear DNA cleavage (Refs 55, 57, 58).

#### Tumour development

PARG is overexpressed in various malignant diseases, such as hepatocellular carcinoma and cancers of the oesophagus, endometrium, colon and rectum, and ovaries (Refs 59–63). The enzyme can promote cancer cell proliferation and metastasis (Refs 59, 60). Mechanistically, PARG dePARylates damage-specific DNA binding protein 1 (DDB1), thereby up-regulating its auto-ubiquitination and decreasing its stability in hepatocellular carcinoma cells (Ref. 59). DDB1 acts as a component of the E3 ubiquitin ligase complex to ubiquitinate Myc for degradation (Ref. 64). The presence of PARG indirectly stabilises Myc and promotes cancer cell proliferation (Ref. 59). In cancerous oesophageal cells, PARG promotes disease progression through activation of the WNT/*β*-catenin signalling pathway; however, the exact molecular mechanism involved remains unclear (Ref. 60). The *O*-GlcNAcylation of PARG results in its destabilisation and reduces DDB1 dePARylation, which decreases c-Myc protein levels, ultimately inhibiting tumour growth (Ref. 65). Moreover, in the presence of PARPi, PARG knockdown significantly induced G2/M cell cycle arrest and cell death (Ref. 63). Simultaneous PARG knockdown and PARP inhibition suppressed the liver metastatic potency of colon carcinoma cells by inhibiting the expression of nuclear factor-kappa B (NF-*κ*B) and activating the phosphatidylinositol 3-kinase/protein kinase B (PI3K/Akt) signalling pathway (Refs 66, 67).

During DNA replication, unligated Okazaki fragments activate PARP1 to generate endogenous S-phase PAR (Ref. 68). PARG must remove these PAR molecules to ensure proper cell cycle progression, as failure to do so will cause DNA damage and cell death (Ref. 69). PARG inhibition leads to the collapse of replication forks in the absence of HR proteins and thereby induces cell death (Refs 70, 71). These findings imply that PARG could be a potential target molecule for treating tumours deficient in HR-mediated repair. However, several reports have shown that the presence of PARG can restrict the growth of tumours and delay their onset (Refs 72, 73), suggesting that PARG has diverse roles depending on the specific cell type or the physiological status of the cells. Therefore, elucidation of the molecular mechanism of PARG in tumourigenesis should provide powerful theoretical support for the use of PARG inhibitors (PARGi) as cancer therapeutics.

## ADP-ribosyl-acceptor hydrolase

The ADP-ribosyl-acceptor hydrolase (ARH) family is an evolutionarily highly conserved structural module, adopting a predominantly *α*-orthogonal bundle architecture, typically consisting of 290–360 residues (Ref. 74). The protein family was first discovered in *Rhodospirillum rubrum*, where the activating factor dinitrogenase reductase-activating glycohydrolase (DraG) was found to reverse dinitrogenase reductase (Fe-protein), an inhibitor of Arg-ADPRylation (Ref. 75). Subsequently, a similar enzyme with comparable properties, designated ARH1, was identified in animal cells (Ref. 14).

Although the three members (ARH1, ARH2 and ARH3) of the ARH family exhibit significant amino acid sequence similarity (Refs 8, 11, 76), ARH1 and ARH3 display distinct substrate specificities: ARH1 cleaves MARylated substrates modified on Arg residues (Ref. 77), whereas ARH3 hydrolyses PAR and *O*-acetyl-ADPr (OAADPr) as well as serine-linked MARylation (Ser-ADPr) (Refs 13, 78–80) (Fig. 1, Table 1). By contrast, ARH2 has not been shown to hydrolyse MAR or PAR, because it lacks the amino acid residues critical for enzymatic activity, which are conserved in both ARH1 and ARH3 (Refs 11, 81, 82).

### ARH1

ARH1, the first ARH family member discovered, was initially isolated from turkey erythrocytes in 1988 and subsequently identified in human, rat and mouse tissues (Refs 77, 83). As a widely expressed cytoplasmic protein, ARH1 is an Arg-MAR hydrolase, meaning that it hydrolyses the *N*-glycosidic bond between ADPr and the Arg guanidino group, thereby releasing ADPr from Arg residue (Ref. 77) (Table 1). Human ARH1 is a 357-amino-acid protein (Ref. 83) (Fig. 2). Its crystal structure in complex with ADP and K^+^ shows *α*-helical protein folds consisting of four helical bundle sub-domains (Refs 84, 85). The enzymatic activities of the rat, mouse and turkey ARH1 molecules are enhanced by dithiothreitol and Mg^2+^, whereas human ARH1 is dithiothreitol independent (Ref. 86). Mg^2+^ is crucial for ARH1 activity, as it helps to properly position the substrate at the catalytic site (Refs 11, 87). Substituting Asp-60 or Asp-61 with Ala, Gln or Asn notably decreased the hydrolase activity of ARH1, indicating the critical role of these amino acid residues at the active site (Ref. 88).

Kato *et al*. (Ref. 89) found that although *Arh1*-knockout (*Arh1*^–/–^) mice were viable, they developed various tumours and were susceptible to *Vibrio cholerae* infections. Moreover, *Arh1* deletion in MEFs and tissues enhanced the sensitivity of the cells and mice to cholera toxin by abolishing the hydrolysis of the endogenously Arg-ADPr-modified *α*-subunit of the intestinal Gs protein (Gs*α*), indicating the crucial role of ARH1 as the primary Arg-specific hydrolase involved in dePARylation (Ref. 89). Additionally, the intestinal mucosa in *Arh1*^–/–^ mice showed a pathological response with an elevated efflux of fluid and electrolytes (Refs 89, 90). *Arh1*^–/–^ and *Arh1*^+/–^ mice are prone to various tumour types, including carcinomas, sarcomas and lymphomas, likely because of accelerated cell proliferation caused by a shortened G1 phase (Ref. 91). Changes in the endogenous oestrogen level in *Arh1*^–/–^ mice appear to be important for lung tumourigenesis, showcasing a gender-specific phenotype (Ref. 92). Furthermore, although the exact mechanism of tumourigenesis in *Arh1*-deficient mice remains largely unknown, the high tumour susceptibility of heterozygous *Arh1*^+/–^ mice highlights the haploinsufficiency of the remaining *Arh1* allele, affecting Arg-MAR hydrolase activity (Refs 91, 93). Other phenotypic observations in *Arh1*-knockout mice include age- and gender-dependent cardiomyopathy characterised by decreased cardiac contractility (Ref. 94). In summary, ARH1 plays a key role in intracellular signal transduction, tumourigenesis and cholera toxin susceptibility, highlighting its significance in cellular functions and disease processes. However, the molecular mechanisms behind these pathological changes remain to be elucidated.

### ARH3

ARH3, a conserved ARH member, is found in various eukaryotes but missing in some eukaryotic taxa, such as the *Nematoda*, *Lepidoptera* and most *Diptera* (including all *Drosophila* species) (Ref. 95). It is widely expressed in many tissues and cells of humans and mice (Ref. 11), being found in the cytoplasm (65%) (Refs 96, 97); the mitochondrial matrix (25%), where its presence depends on an MTS (Refs 96, 98); and the nucleus (10%), despite lacking a nuclear localisation signal (Ref. 97) (Table 1). The nuclear presence of ARH3 may vary depending on the cell type, because it has been observed in mouse brain tissue and MEFs but not in HepG2 cells (Refs 11, 97). Nonetheless, ARH3 may participate in the regulation of mitochondrial functions involving sirtuin (SIRT) 3, SIRT4 and SIRT5, given that these enzymes are located in the mitochondrial matrix and conduct NAD^+^-dependent deacetylase activity, generating OAADPr (Ref. 99).

Human ARH3 has 40% sequence similarity and 20% identity with human ARH1 (Ref. 100). However, unlike ARH1, which cleaves only MARylated residues, ARH3 can degrade long PAR polymers, cleaving the Ser-ADPr linkage, and also hydrolyse OAADPr (Refs 11–13). The ARH3 gene, which comprises 6 exons, encodes a 363-amino-acid protein that includes a predicted N-terminal MTS (Refs 78, 98) (Fig. 2). Mueller-Dieckmann *et al*. (Ref. 78) determined the crystal structure of ARH3 (with a 16-amino-acid truncation at the N terminus), in the presence and absence of ADP, at a resolution of 1.6 angstroms and found that the enzyme has an all-*α*-helical fold form, with the active-site crevice flanked by two Mg^2+^ ions surrounded by highly conserved amino acids. ARH3-catalysed reactions are significantly stimulated by Mg^2+^ and enhanced by dithiothreitol (Refs 79, 80, 87). A conformational change in the Glu-41-containing flap motif of ARH3 enables specific substrate recognition and cleavage (Ref. 101). Asp-77 and Asp-78 mutations in ARH3 abolish its ADPr hydrolase activity but do not affect its binding to ADPr (Ref. 11). Likewise, Gly-115, Ser-148, Tyr-149, His-182, Asp-314 and Thr-317 mutations abolish the enzyme's ability to hydrolyse ADP-ribosylated substrates (Ref. 12). Human ARH3 exhibits a micromolar affinity for free ADPr and efficiently conducts deADPRylation of PARylated but not MARylated proteins (Ref. 78). The activities of both ARH1 and ARH3 are inhibited by ADPr (Refs 11, 102). Additionally, ARH3 is inhibited by ADP-(hydroxymethyl) pyrrolidinediol (ADP-HPD) (Ref. 87). Although ARH1 and ARH3 share high structural similarity, they exhibit different modes of ligand binding. The elucidation of their structures has provided insights into their observed selectivity of *α*-1100-linked substrates (Ref. 87).

PARG is the primary enzyme responsible for breaking down PAR chains, hydrolysing both endo- and exo-glycosidic bonds, whereas ARH3 is unable to cleave branched PAR chains and instead catalyses the exo-glycosidic hydrolysis of linear PAR forms, generating free ADPr molecules and short PAR chains (Refs 102, 103). As ARH3 and PARG are the main members of PAR-degrading enzymes, their simultaneous inhibition causes cell death owing to the excessive accumulation of PAR and PARylated proteins that may disturb cellular activities (Ref. 26). Additionally, the loss of either PARG or ARH3 increases cellular resistance to PARPi. In the presence of PARPi, PARG deletion reduces PARP1–DNA complex formation, prevents unrestricted replication fork progression and partially rescues recruitment of the scaffold protein X-ray repair cross complementing 1 to sites of DNA damage, thereby leading to a reduction in PARPi-induced DNA damage and cell death (Ref. 104). Although the feasibility of ARH3 loss as a PARPi-resistant mechanism remains unclear, PARPi have therapeutic potential for treating neurodegeneration caused by ARH3 deficiency (Ref. 26). Because ARH3 exhibits less than 10% of the catalytic activity of PARG, it possibly acts as a backup for PARG to remove the excessive PAR moieties that are produced under stress conditions (Refs 11–13). The Ser-ADPRylation catalysed by the cooperative activities of PARP1/2 and histone PARylation factor 1 is the primary form of ADPRylation in the DNA damage response (DDR) of cells (Refs 13, 105). Therefore, as the only enzyme that can specifically hydrolyse Ser-ADPr, ARH3 plays an important role in DNA repair.

*Arh3*^–/–^ mutant mice are viable but susceptible to cerebral ischaemia reperfusion (Ref. 106). Interestingly, in the absence of both ARH3 and PARG, PAR accumulates, which in turn promotes parthanatos (Refs 57, 97). Thus, collaboration of these two PAR erasers is important for regulating the cell response to oxidative stress-mediated parthanatos. This is consistent with the observation that ARH3-deficient mice appeared to be normal under physiological conditions until environmental stress insults were encountered, which is in contrast to a total loss of PARG leading to embryonic death (Refs 41, 106). The distinct functions of ARH3 and PARG perhaps rely on their sub-cellular localisations, PARylated substrates (amino acid residues), stressors and cell-type specificity. ARH3 deficiency is associated with neurological degeneration in humans (Ref. 74). The enzyme plays a critical role in preventing stress-induced PARP1-dependent neuronal cell death through its PAR-degrading activity, which correlates with the clinical presentation of ARH3-deficient individuals, whose phenotypes appear to be induced by environmental stress (Refs 106–111). These observations indicate that ARH3 likely participates in an important regulatory pathway to prevent parthanatos in neurons by maintaining PAR homeostasis.

## Macrodomain-containing proteins MacroD1 and MacroD2

The macrodomain fold, which is a compact globular-shaped structure of approximately 25 kDa size, is widely distributed among all life forms (including viruses) and evolutionarily conserved (Ref. 112). It is present in MacroD1, MacroD2 and TARG1, which possess the ability to hydrolyse MAR from proteins (Ref. 113). In humans, MacroD1 and MacroD2 are highly similar members of the MacroD-type class. Although both were initially identified as deacetylases of OAADPr (Ref. 114), they were later found to hydrolyse MAR, specifically targeting the ester bond formed by ADP-ribosylated Asp/Glu residues (Refs 15, 115) (Fig. 1). MacroD1 is predominantly localised to the mitochondrial matrix (Refs 116, 117), whereas MacroD2 is distributed in the cytosol and nucleus (Refs 115, 118) (Table 1). The mRNA of MacroD1 is expressed in various tissues, with a high level in skeletal muscle (Ref. 119), whereas the mRNA of MacroD2 is expressed in multiple tissues during the embryonic period, including the liver, brain, lung, thymus, heart, kidney, etc., highly expressed in the brain neurons at embryonic and adult stage (Refs 120, 121). According to these reports, the distinct localisation of the macrodomain proteins suggests that they have unique roles to play, despite their structural similarities.

The approximately 35 kDa MacroD1 protein is encoded by 11 exons. Analysis of its crystal structure revealed that it comprises an N-terminal region (91–136 amino acids, encoded by exons 1–3), a macrodomain region (151–322 amino acids, encoded by exons 3–9) (Ref. 114) and an MTS (1–85 amino acids, encoded by exon 1) (Ref. 115) (Fig. 2). MacroD2 is an approximately 47 kDa protein that is encoded by 17 exons. Similar to MacroD1, the crystal structure of MacroD2 constitutes an N-terminal region (7–47 amino acids, encoded by exons 1–2) and a macrodomain region (78–228 amino acids, encoded by exons 3–9) (Ref. 122). In both MacroD1 and MacroD2, the N-terminal region is arranged as helical segments and a short *β*-sheet, while the macrodomain region resembles a canonical macrodomain fold, being composed of a three-layered *α*–*β*–*α* sandwich with a central six-stranded *β*-sheet (Refs 114, 122, 123). MacroD1 and MacroD2 have similar catalytic mechanisms for mediating ADPr hydrolysis. Upon either enzyme binding to ADPr, structural re-arrangement occurs to ensure the correct positioning of the substrate (Refs 115, 122, 123). Meanwhile, the hydrogen bond network formed among water molecules, ADPr *α*-phosphates and other elements is responsible for the precise positioning of water molecule in the catalytic pocket (Ref. 123). Then, ADPr *α*-phosphates activate the water molecule, allowing it to make a nucleophilic attack on the distal ribose C1" atom of ADPr, thereby cleaving the glycosidic bond between this atom and the acceptor Asp/Glu residue (Refs 115, 123).

MacroD1, also known as leukaemia-related protein 16, is primarily localised in the mitochondrial matrix (Ref. 119). Nonetheless, it has been associated with several nuclear functions, including the activation of NF-*κ*B signalling, binding and regulation of oestrogen and androgen receptors, and counteracting of PARP7-mediated MARylation (Refs 124–127). MacroD1 has also been proposed as a negative regulator of the insulin signalling pathway through its down-regulation of insulin receptor substrate protein 1 (Ref. 128). Additionally, MacroD1 expression and gene fusions have been implicated in the tumourigenesis of leukaemia and breast, gastric, liver, lung and colorectal cancers (Refs 129–132). Crawford *et al*. (Ref. 133) reported that *Macrod1*-knockout mice were viable and fertile but exhibited a female-specific motor coordination defect. Loss of MacroD1 in rhabdomyosarcoma cells resulted in mitochondrial fragmentation (Ref. 119). MacroD2 has been shown to undergo phosphorylation by ataxia-telangiectasia-mutated kinase in response to DNA DSBs and to be involved in reversing the ADPRylation of glycogen synthase kinase 3 beta, a key kinase in the WNT-mediated signal transduction pathway (Refs 118, 134). However, the role of MacroD1 in DNA damage repair has yet to be fully elucidated.

MacroD2 is highly expressed in neuronal tissues and cells, which supports the observation that its mutation leads to a neurodegenerative phenotype (Refs 135, 136). The *Macrod2* gene locus is linked to several neurological syndromes (e.g. autism, attention-deficit hyperactivity disorder and schizophrenia), which correlates with the notable expression of the protein in neurons during brain development (Ref. 120). According to Crawford *et al*. (Ref. 133), *Macrod2*-knockout mice exhibited age-dependent hyperactivity along with a gait resembling bradykinesia, but neither *Macrod1-* nor *Macrod2*-knockout mice showed any defects in short-term working memory or attention span.

## Terminal ADP-ribose glycohydrolase

Terminal ADP-ribose glycohydrolase (TARG1), also called *O*-acyl-ADP-ribose deacylase 1 (OARD1), which has been well characterised as an enzyme that hydrolyses MARylation, is ubiquitously expressed in various tissues and found in both the nucleus and cytoplasm, with high levels in the nucleolus (Ref. 119). It was initially found to be enriched in chronic lymphocytic leukaemia cells and subsequently demonstrated to possess deacylation activity, producing free ADPr from OAADPr, OPADPr (*O*-propionyl-ADPr) and OBADPr (*O*-butyryl-ADPr) deacylation (Refs 137, 138). Additionally, TARG1 has been reported to remove ADPr units from MARylated PARP1, which is auto-modified at the Asp/Glu residues (Ref. 16) (Fig. 1, Table 1).

The approximately 17 kDa TARG1 protein, which is encoded by 8 exons, consists of a macrodomain (1–152 amino acids, encoded by exons 4–8) that contains only the core domain and lacks the N- and C-terminal extension structures common in other macrodomains (Ref. 138) (Fig. 2). Similar to other macrodomains, the TARG1 macrodomain consists of a three-layer *α*–*β*–*α* sandwich containing a six-stranded *β*-sheet flanked by four *α*-helical elements (Refs 16, 138). A hydrophobic pocket that can embed an adenosine moiety is formed by Leu-21 and Phe-22 on the *β*_1_–*β*_2_ loop, Ile-44 and Leu-47 on the *β*_2_–*α*_1_ loop, and Pro-118, Tyr-150 and the C-terminal Leu-152 residue (Ref. 138). The active centre located in the vicinity of the distal ribose is composed of Ser-35, Lys-84 and Asp-125 (Refs 16, 138). The hydroxyl group of Ser-35 forms a hydrogen bond with the carbonyl oxygen of an ester, polarising the carbonyl bond (Ref. 138). Lys-84 forms a covalent intermediate with ADPr through the Amadori re-arrangement mechanism, following which Asp-125 hydrolysis and ADPr release ensue (Ref. 16).

Because research on TARG1 is still in its infancy, the physiological role of this enzyme in organisms remains largely unknown. TARG1 deficiency caused the senescence of U2OS and 293T cells and decreased the proliferation of 293T but not HeLa cells (Refs 16, 139). TARG1 is enriched in the nucleolus, where its loss leads to an increase in the number of nucleoli and hyper-active transcription (Ref. 119), implying its importance in nucleolar homeostasis and function. Moreover, it can shuttle rapidly between the nucleoplasmic and nucleolar compartments (Ref. 139). When DNA damage occurs, TARG1 moves quickly from the nucleolus into the nucleoplasm where it interacts with the PAR units enriched at the DNA damage site. The deletion of TARG1 rendered cells sensitive to DNA damage agents, topoisomerase II and ataxia telangiectasia and Rad3-related (ATR) inhibition and also damaged HR repair (Refs 16, 139, 140), suggesting that the enzyme is involved in DNA repair. *TARG1* mutations which result in truncated proteins without catalytic activity have been reported to cause neurodegeneration in humans (Ref. 16). Whether this is due to the cytotoxicity of the truncated protein or an imbalance of MARylation remains unclear. Given these findings, the molecular mechanism underlying the roles of TARG1 in nucleolar homeostasis and the DDR remains to be studied.

ADPRylation occurs not only in proteins but also in DNA and RNA (Refs 141–144). DNA ADP-ribosyl transferase (DarT), a DNA-modifying PARP-like bacterial toxin, can be released by bacteria into human host cells where it targets single-stranded DNA during DNA replication to induce DNA ADPRylation, thereby triggering the DDR (Refs 145–147). One study found that TARG1 could eliminate the toxic DNA ADPRylation induced by ectopic DarT expression in host cells, thereby ensuring normal cell proliferation (Ref. 145). This finding suggests that TARG1 plays a protective role against bacterial toxins analogous to DarT, similar to the effect of ARH1 against cholera toxin (Ref. 89). In ovarian cancer cells, the PARP14-mediated site-specific MARylation of receptor for activated C kinase 1 (RACK1) promoted the assembly of stress granules in response to external stimuli (Ref. 148). RACK1 is a scaffold protein of the 40S ribosome subunit and an essential member of stress granules (Refs 149, 150). TARG1 erases MARylation and dissociates RACK1 from stress granules (Ref. 148), implying that it may serve as a potential molecular target for the treatment of ovarian cancer.

MacroD1, MacroD2 and TARG1 are the most extensively characterised macrodomain-containing hydrolases. Although they belong to the same family and hydrolyse MAR, they target different substrates through distinct molecular mechanisms (Ref. 1). The physiological functions of these three enzymes remain elusive, and further research is necessary to comprehend their precise role in MAR hydrolysis. For instance, the nuclear function of MacroD1 and the reason behind its predominantly mitochondrial localisation warrant further study. Additional much-needed investigations include whether these enzymes exhibit substrate specificity, which depends on different MARylation sites and the physiological status of the cells and tissues.

## Inhibitors of PAR hydrolases

PARylation and ADPr metabolism play fundamental roles in DNA repair to maintain genome stability, and the targeting of PARylation synthesizing PARP enzymes within this pathway has shown therapeutic potential (Ref. 19). Various PARPi have been developed for cancer treatment, with olaparib, niraparib and rucaparib already approved for clinical use against specific tumour types (Refs 17, 151, 152). Recently, PARGi have also generated immense interest in relation to the pharmacological intervention of different maladies and emerged as promising targets for a variety of cancers and other diseases (Refs 31, 153–158).

Proflavine, ethacridine, ellipticine, daunomycin and tilorone, which are all DNA intercalators, were found to inhibit PARG activity, which may be indirectly caused by their insertion into the DNA molecule, resulting in the release of inhibitory histones (Ref. 159). However, owing to the high cytotoxicity of these DNA intercalators, a second generation of PARGi based on tilorone (viz. GPI16552 and GPI18214) were developed. GPI16552 significantly reduced the cerebral infarction volume in a rat model of focal cerebral ischaemia and attenuated the inflammatory response and tissue damage caused by spinal cord trauma (Refs 160, 161). Treatment with GPI18214 attenuated zymosan-induced multiple organ failure in mice, reducing peritonitis in the animals as well as their mortality rate (Ref. 162). GPI16552 and GPI18214 can mitigate splanchnic artery occlusion as well as reperfusion- and dinitrobenzene sulphonic acid-induced intestinal injury (Refs 163, 164). PARG inhibition significantly reduces the expression of the pro-inflammatory cytokines tumour necrosis factor alpha (TNF*α*) and interleukin 1 beta (IL1*β*) as well as neutrophil infiltration (Refs 161, 162, 164), which account for the protective effect of PARGi.

Tannins, particularly gallotannins and ellagitannins, are naturally occurring polyphenol compounds with PARG-inhibiting activity, which is mediated through their competitive binding to PAR with PARG (Ref. 165). With regard to the oligomeric forms of ellagitannin, the dimer nobotanin B exhibits stronger PARG inhibitory activity than the trimer nobotanin E and the tetramer nobotanin K (Ref. 165). Gallotannins and nobotanin B significantly reduce the oxidative death of astrocytes and neurons by accumulating PAR and thus slowing its turnover, thereby blocking PARP1-mediated cell death (Ref. 166). PARG inhibition by gallotannin decreased ischaemic brain damage and significantly ameliorated infarct formation and neurological deficits in rats (Ref. 167), indicating that PARGi have a neuroprotective function. Galloyl-glucose derivatives based on gallotannin have potent PARG inhibitory activity, albeit with low cell permeability, reducing PARP1-dependent cell death to some extent (Ref. 168). ADP-HPD is an amino-ribose analogue of ADPr, in which half maximal inhibitory concentration (IC_50_) reaches the nanomolar grade (Ref. 169). However, because of its low cell permeability, it is mostly used in *in vitro* studies to elucidate the structure and catalytic activity of PARG. Pargamicin, a cyclic peptide isolated from an *Amycolatopsis* sp. fermentation product, has weak PARG inhibitory activity (Ref. 170). With the advancement of identification methods, a series of PARGi xanthene compounds (Eosin Y and Phloxin B) (Ref. 171), salicylanilide (Ref. 172), RBPIs (rhodanine-based PARGi) (Ref. 173) and phenolic hydrazide hydrazones (Ref. 174) with IC_50_ values in the micromolar range have been reported. Recently, newly developed quinazolinedione sulphonamides (PDD00017273 and PDD00017238) and a thio-xanthine/methylxanthine derivative (JA2131) have achieved PARG inhibitory activity comparable to that of ADP-HPD (Refs 175–177). PDD00017273 is effective against pancreatic ductal adenocarcinoma cells carrying the BRCA2 mutation but has no effect on breast cancer cell lines with BRCA1 or BRCA2 mutations (Refs 175, 178), implying that its PARG inhibitory effect is potent for only certain types of cancer. JA2131 inhibits PARG activity by binding to the enzyme's adenine-binding pocket, thereby killing cancer cells by promoting PAR accumulation and *γ*H2AX foci in the nucleus (Ref. 177). COH34, a small molecule with anti-tumour effects, can bind specifically to PARG to inhibit its activity at sub-nanomolar concentrations, resulting in extended PARylation, which blocks DNA repair and inhibits the growth of cancer cells with DNA repair defects (Ref. 179). Recently, the company IDEAYA Biosciences has conducted a clinical trial of PARGi for patients bearing tumours harbouring HR deficiency (HRD), including ER+, Her2-, HRD breast cancer and HRD ovarian cancer (https://www.ideayabio.com/pipeline/). Deploying PARGi or targeting dePARylation enzymes represents a novel clinical approach to fighting human diseases, including cancer.

As mentioned previously, ARH3 is the only known ADPr hydrolase to remove ADPr from serine residues, which is the major type of ADPRylation of histones during DNA damage repair. Thus, ARH3 inhibitors are currently being developed as a chemotherapeutic strategy for tumour suppression (Ref. 180). In contrast to the PARP family, which consists of multiple members, the PARG family comprises only a single member. This characteristic lends the advantage of specificity to targeted PARG therapy and helps to overcome the issue of tumour cell resistance to PARPi (Ref. 113). Furthermore, PARP1 is highly abundant in cells, with an estimated 10^6^ molecules per cell (Ref. 181). By contrast, each cell contains approximately 2000 PARG molecules (Ref. 182), suggesting that PARG may offer enhanced potency and cell-type specificity for cancer treatment. Of note, ADPr homeostasis involves not only dePARylation but also deMARylation. Therefore, the identification or development of inhibitors targeting both these processes could have a profound impact on cancer research. Although many PARGi have been reported, whether they also target other enzymes or proteins relevant to ADPr degradation has not been well studied.

The successful Food and Drug Administration (FDA) and European Medicines Agency (EMA) approvals of the clinical use of PARPi for cancer treatment have gained momentum (Ref. 183). However, owing to the issue of cancer resistance to PARPi in some patients, alternative therapeutic strategies need to be explored. Targeting PARG and other ADPr hydrolases to modulate PAR metabolism may represent a novel approach for cancer chemotherapy. The elucidation of the X-ray crystal structure of PARG (Refs 35, 37, 184) is considered a significant achievement in ADPr hydrolase research, as the findings have the potential to greatly expedite the development of novel and potent PARGi, which will undoubtedly contribute to the creation of therapeutic drugs that target ADPr-degrading enzymes.

## Perspectives

ADPRylation is a prevalent PTM that regulates various cellular pathways and is involved in disease pathogenesis processes, including the DDR, cell death, transcription, chromatin remodelling, neurodegenerative disorders and inflammatory reactions (Ref. 185). Unravelling the molecular and biological functions as well as substrate specificities of the synthetising and degrading enzymes of PARylation and MARylation will aid in the elucidation of their roles in specific signalling pathways and the identification of potential targets for clinical applications. Different ADPr hydrolases have distinct substrates and sub-cellular localisations, working collaboratively to coordinate the removal of ADPRylation modifications. PARG predominantly degrades (long and branched) PAR chains but has limited activity on short PAR polymers, indicating that other erasers, such as ARH3, may act in concert with the hydrolases to erase ADPr efficiently (Ref. 184). Complete reversal of MARylation is performed solely or in concert by MacroD1, MacroD2, TARG1, ARH1 and ARH3. However, despite great efforts, the specific similarities and differences in the hydrolysis process catalysed by these enzymes, as well as the precise nature of their regulation, remain poorly understood. Given the importance of PARylation and MARylation in diverse signalling networks, further investigations into the roles of the erasers of these PTM processes in different cell types and tissues, as well as in cancerous versus non-cancerous cells, are needed. These studies will not only help to elucidate the biological roles of the ADPr hydrolases but also provide valuable reference information for the future development of chemotherapeutic strategies against PAR homeostasis-related diseases, including cancer.
